# Validation of Pre-/Post-TACE-Predict Models among Patients with Hepatocellular Carcinoma Receiving Transarterial Chemoembolization

**DOI:** 10.3390/cancers14010067

**Published:** 2021-12-23

**Authors:** David Sooik Kim, Beom Kyung Kim, Jae Seung Lee, Hye Won Lee, Jun Yong Park, Do Young Kim, Sang Hoon Ahn, Seung Up Kim

**Affiliations:** 1 Department of Internal Medicine, Yonsei University College of Medicine, Seoul 03722, Korea; powerof1004@gmail.com (D.S.K.); BEOMKKIM@yuhs.ac (B.K.K.); SIKARUE@yuhs.ac (J.S.L.); LORRY-LEE@yuhs.ac (H.W.L.); DRPJY@yuhs.ac (J.Y.P.); DYK1025@yuhs.ac (D.Y.K.); AHNSH@yuhs.ac (S.H.A.); 2 Institute of Gastroenterology, Yonsei University College of Medicine, Seoul 03722, Korea; 3 Yonsei Liver Center, Severance Hospital, Yonsei University Health System, Seoul 03722, Korea

**Keywords:** hepatocellular carcinoma, risk, prediction, prognosis, transarterial chemoembolization

## Abstract

**Simple Summary:**

Transarterial chemoembolization (TACE) is used to treat patients with intermediate stage hepatocellular carcinoma (HCC). However, models to accurately predict survival are lacking. The aim of our retrospective study was to attempt to validate the prognostic performance of the newly proposed Pre- and Post-TACE-Predict models with Korean patients. In our study of 187 patients with HCC who underwent TACE, there was no significant difference between the Pre- and Post-TACE prediction models in HCC patients. Additionally, simple scoring prognosis prediction models performed similarly to or better than the Pre- and Post-TACE-Predict models in our study. Thus, simple scoring prognosis prediction models such as modified hepatoma arterial embolization prognostic (mHAP)-II and SNACOR may be useful in assessing the TACE treatment survival over the Pre- and Post-TACE-Predict models in patients with HCC.

**Abstract:**

This study attempted to validate the prognostic performance of the proposed Pre- and Post-TACE (transarterial chemoembolization)-Predict models, in comparison with other models for prognostication. One-hundred-and-eighty-seven patients with HCC who underwent TACE were recruited. Regarding overall survival (OS), the predictive performance of the Pre-TACE-Predict model (one-year integrated area under the curve (iAUC) 0.685 (95% confidence interval (CI) 0.593–0.772)) was better than that of the Post-TACE-Predict model (iAUC 0.659 (95% CI 0.580–0.742)). However, there was no significant statistical difference between two models at any time point. For comparison between models using pre-treatment factors, the modified hepatoma arterial embolization prognostic (mHAP)-II model demonstrated significantly better predictive performance at one year (iAUC 0.767 (95% CI 0.683–0.847)) compared with Pre-TACE-Predict. For comparison between models using first TACE response, the SNACOR model was significantly more predictive at one year (iAUC 0.778 (95% CI 0.687–0.866) vs. 0.659 (95% CI 0.580–0.742), respectively) and three years (iAUC 0.707 (95% CI 0.646–0.770) vs. 0.624 (95% CI 0.564–0.688), respectively) than the Post-TACE-Predict model. mHAP-II and SNACOR may be preferred over the Pre- and Post-TACE-Predict models, respectively, considering their similar or better performance and the ease of application.

## 1. Introduction

Hepatocellular carcinoma (HCC) is the third most common cause of cancer-related death [1]. In cases of early-stage diagnosis, curative treatments for HCC, including surgical resection, orthotopic liver transplant, or local ablation, have been shown to be feasible. In contrast, patients diagnosed with intermediate or advanced HCC are generally treated with palliative modalities, including transarterial chemoembolization (TACE) or systemic chemotherapy, such as sorafenib, regorafenib, and atezolizumab/becacizumab [2,3]. Among these, TACE, which is based on induction of focal ischemia with local delivery of chemotherapeutic agents [4], has been shown to improve survival more than supportive care in randomized controlled trials [5,6]. Thus, current guidelines recommend TACE as the standard treatment for patients with intermediate stage (Barcelona Clinic Liver Cancer (BCLC) stage B), multi-nodular HCC who are not candidates for curative treatment [7,8].

However, several unresolved issues remain. First, there is widespread use of TACE outside of recommended guidelines; more specifically, early stage or advanced stage with portal vein tumor invasion, which hinders an accurate assessment of long-term clinical outcomes after TACE [9,10]. Additionally, due to the heterogeneity of patients with BCLC stage B HCC, the decision-making process regarding treatment modalities tends to depend on the physician’s discretion rather than the simplified algorithm based on practice guidelines [11]. As such, many studies have attempted to further sub-classify such patients to guide the optimal treatment strategy and prognostication after TACE treatment [12,13]. Among the prognosis prediction models developed to date, the hepatoma arterial embolization prognostic (HAP) score consists of a point system according to tumor size, alpha-fetoprotein (AFP), bilirubin, and albumin [14]. This was further improved by Park et al., with the addition of tumor number, as the modified HAP-II (mHAP-II) [15]. Although both predictors were effective and easy to use, one limitation was that they did not address the treatment response after TACE, given that it may substantially alter the final outcome of patients with HCC. Therefore, many studies have attempted to develop models that include HCC response to TACE. For example, “SNACOR,” developed by Kim et al., is a prognosis prediction model that accounts for radiological response after the first TACE session [16]. More recently, Han et al. [17] proposed an individualized TACE-specific prognosis prediction model using widely available clinical features and response to first TACE therapy—more specifically, Pre-TACE-Predict and Post-TACE-Predict models—both of which demonstrated superior predictive performance compared with HAP and mHAP-II scores.

In the present study, we attempted to externally validate the prognostic performance of the newly proposed Pre-TACE-Predict and Post-TACE-Predict models compared with other prognosis prediction models in an independent cohort of Korean patients with HCC undergoing TACE.

## 2. Materials and Methods

### 2.1. Patients

**Figure 1:**
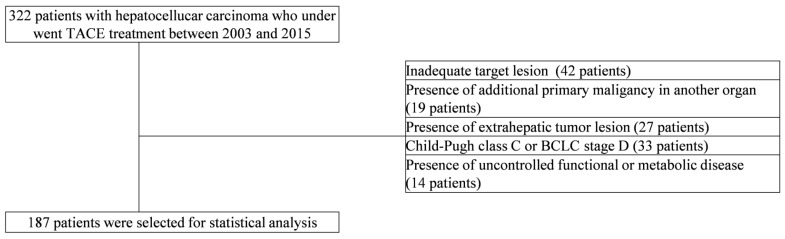
Flow of selecting the study population. A total of 322 patients with hepatocellular carcinoma from Yonsei University Health System who were treated with TACE between 2003 and 2015 were initially included in our study. After the exclusion of 135 patients due to our exclusion criteria, a total of 187 patients were ultimately included for the statistical analysis. TACE, transarterial chemoembolization.

Data from patients with treatment-naïve HCC, who underwent TACE between 2003 and 2015 at the Severance Hospital, Yonsei University College of Medicine (Seoul, Korea), were included in the present study. Exclusion criteria are reported in Figure 1. HCC was diagnosed using histological or radiological methods according to current practice guidelines [4,5]. According to the etiology of HCC, patients were categorized as follows: hepatitis B virus (HBV); hepatitis C virus (HCV); and other, which covered HCC arising from chronic liver diseases other than HBV and/or HCV.

The study was conducted according to the guidelines of the Declaration of Helsinki. Given the retrospective nature of the study and the use of anonymized patient data, requirements for informed consent were waived.

### 2.2. TACE Procedure and Assessment of Treatment Responses

Angiography of the hepatic artery and superior mesenteric artery was performed to confirm portal vein patency, vascular anatomy, and tumor vascularity. TACE was performed using 5 mL of iodized oil contrast medium (lipiodol), and 50 mg of either adriamycin or cisplatin (2 mg/kg body weight). The mixture was infused selectively at the subsegmental or segmental branch of the feeding arteries, followed by embolization using gelatin sponge particles. Dynamic liver computed tomography (CT) was used to detect residual tumors and, if detected, sequential TACE procedures were scheduled at 6–8 week intervals when extrahepatic metastases, critical portal vein invasion, and deterioration in clinical status or laboratory values were not detected.

Treatment response was radiologically assessed 4 weeks after the initial TACE procedure according to the modified Response Evaluation Criteria in Solid Tumors (mRECIST), criteria, as described by Lencioni et al. [18]. The four mRECIST categories included complete response (CR), partial response (PR), stable disease (SD), and progressive disease (PD). Viable tumors were assessed using the uptake of contrast materials during the arterial phase of dynamic CT or magnetic resonance imaging. Retention of iodized oil and necrotic lesions without intra-tumoral arterial enhancement was regarded to be a necrotized tumor foci. CR was defined as complete disappearance of measurable lesions, whereas PR was defined as a 30% decrease from baseline. PD was defined as a 20% increase from the baseline, and SD was defined as the value between PD and PR. Two experienced radiologists with considerable experience read the examinations, both blinded to each other’s results and clinical data. Then, ultimately, final classifications made by consensus between 2 observers were adopted for analysis.

### 2.3. Calculation of Prognosis Prediction Models after TACE

The HAP, mHAP-II, and SNACOR models were used to evaluate the prognosis among patients with HCC after TACE. Detailed scoring and classification are summarized in Table 1. Calculations for Pre-TACE-Predict and Post-TACE-Predict models and cutoffs are described by Han et al. [17] and are as follows:

Pre-TACE-Predict linear predictor = 0.313 × tumor number (0 = solitary, 1 = multifocal) + 1.252 × log_10_ tumor size (cm) + 0.230 × baseline log_10_ AFP (ng/mL) − 0.0176 × baseline albumin (g/L) + 0.458 × baseline log_10_ bilirubin (μmol/L) + 0.437 × VI (0 = no, 1 = yes) + 0.149 × HBV (0 = no, 1 = yes) + 0.333 × alcohol (0 = no, 1 = yes) + 0.211 × other cause if not HCV/HBV/alcohol (0 = no, 1 = yes)

Linear predictor = 0.207 × tumor number (0 = solitary, 1 = multifocal) + 1.129 × log_10_ tumor size (cm) + 0.147 × baseline log_10_ AFP (ng/mL) + 0.750 × baseline log_10_ bilirubin (μmol/L) + 0.447 × VI (0 = no, 1 = yes) + 0.469 × PR (0 = no, 1 = yes) + 1.143 × SD (0 = no, 1 = yes) + 1.354 × PD (0 = no, 1 = yes)

where CR is the reference group for mRECIST.

Pre-TACE-Predict risk category cutoffs were ≤0.94, >0.94 to ≤1.47, >1.47 to ≤2.10, and >2.10 for risk categories 1, 2, 3, and 4, respectively. Post-TACE-Predict risk category cutoffs were ≤1.82, >1.82 to ≤2.49, >2.49 to ≤3.37, and >3.37 for risk categories 1, 2, 3, and 4, respectively.

### 2.4. Statistical Analysis

Categorical variables are expressed as number and percentage (n [%]), and continuous variables are expressed as median (interquartile range [IQR]). Overall survival (OS) was calculated as the difference between the date of TACE treatment and the date of death or the last follow-up. If overall survival of 50% was not reached, values for 95% confidence interval (CI) were labeled as not applicable (NA). The Shapiro–Wilk test was used to assess for normality of distribution. Survival time was calculated using the Kaplan–Meier method. The primary endpoint of this study is the predictive performance of the prognosis prediction models for OS which is calculated using Heagerty’s integrated area under the curve (iAUC) from 1000 bootstrap resamples. For comparison of the primary outcome, if the 95% confidence interval (CI) for the difference between models at each time point included zero, there was no statistical difference between the performances of the models. In addition, regarding detailed specifications of Pre- and Post-TACE-Predict score for prognostication, we calculated Harrell’s C index, Gönen and Heller’s K, Royston-Sauerbrei’s R2D, Akaike information criterion, homogeneity measured using the likelihood ratio chi-squared test, and discriminatory ability measured using the linear trend chi-squared test [19,20]. Statistical analysis was performed using SPSS version 25.0 (IBM Corporation, Armonk, NY, USA) and R (V.4.0.1, http://cran.r-project.org/; accessed on 15 September 2021). Differences with *p* < 0.05 were considered to be statistically significant.

## 3. Results

### 3.1. Baseline Characteristics

After the exclusion of 135 patients, 187 HCC patients who underwent TACE treatment were included in the present study (Figure 1). Baseline demographics of the included patients are summarized in Table 2. The median age of the study population was 59 years, with 136 (72.7%) males and 51 (27.3%) females. The median OS of the entire cohort was 33.4 (IQR 11.7–81.7) months. The main causes of HCC in this patient population included HBV, HCV, and others, accounting for 127 (67.9%), 31 (16.6%), and 29 (15.5%) patients, respectively.

### 3.2. Patient Distributions According to the Prognosis Prediction Model before and after the First TACE

The distribution of patients according to the Pre-TACE-Predict model was 4 (2.1%), 40 (21.4%), 78 (41.7%), and 65 (34.8%) for risk groups 1, 2, 3, and 4, respectively (Table 2).

After the first TACE, the treatment response according to the mRECIST criteria was 28 (15.0%), 81 (43.3%), 41 (21.9%), and 37 (19.8%) for CR, PR, SD, and PD, respectively. Accordingly, the distribution of patients using the Post-TACE-Predict model was 31 (16.6%), 51 (27.3%), 27 (14.4%), and 78 (41.7%) for risk groups 1, 2, 3, and 4, respectively (Table 3).

### 3.3. OS According to Radiological Response after the First TACE

**Figure 2:**
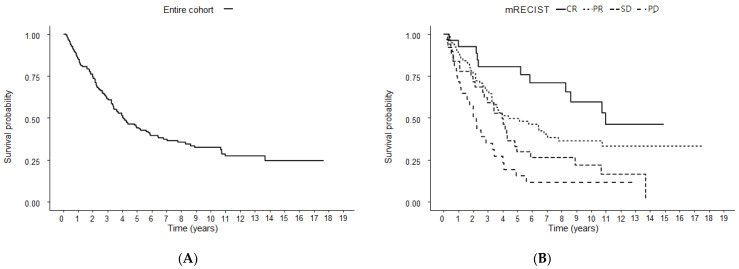
Kaplan-Meier survival curve from baseline (**A**) and after 1st TACE (**B**). TACE, transarterial chemoembolization. Modified Response Evaluation Criteria in Solid Tumors. Complete response, CR; partial response, PR; stable disease SD; and progressive disease; PD.

The median OS for all patients undergoing TACE treatment was 49.2 months (Figure 2). Patients who demonstrated a better response according to mRECIST criteria experienced a longer median OS, with a median OS of 131.9 (95% CI 99.3–NA), 52.4 (95% CI 39.3–128.9), 47.6 (95% CI 32.9–70.9), and 26.5 (95% CI 14.2–48.3) months for CR, PR, SD, and PD, respectively (Figure 2).

Among the overall population, the median time to progress was 8.7 months (95% CI 7.253–10.147). Among patients who achieved CR after 1st TACE, the treatment response was maintained for the median duration of 16.8 months (IQR 7.1–32.1).

### 3.4. OS According to the Pre- and Post-TACE-Predict Models

**Figure 3:**
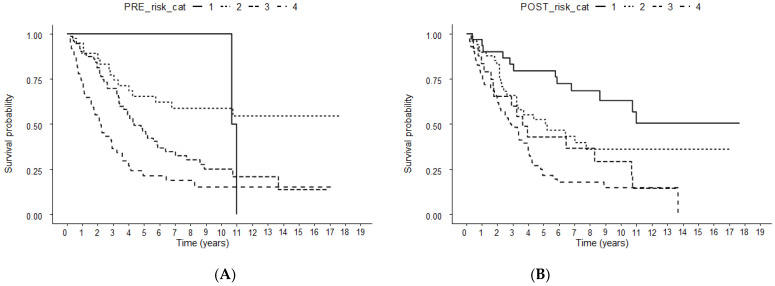
Kaplan-Meier curves according to stratification by Pre- (**A**) and Post-TACE-Predict (**B**) score. TACE, transarterial chemoembolization.

Similarly, patients with a lower risk when stratified according to Pre-TACE-Predict and Post-TACE-Predict models demonstrated a better median OS: 130.0 (95% CI 128.2–NA), not-reached (95% CI 69.0–NA), 52.4 (95% CI 41.0–76.9), and 26.8 (95% CI 19.4–43.3) months for risk groups 1, 2, 3, and 4, respectively, according to the Pre-TACE-Predict model and not-reached (95% CI 103.6–NA), 61.5 (95% CI 39.3–NA), 43.6 (95% CI 21.4–NA), and 34.7 (95% CI 25.6–48.3) months for risk groups 1, 2, 3, and 4, respectively, according to the Post-TACE-Predict model (Figure 3).

### 3.5. Comparison of the Predictive Performance between Pre- and Post-TACE-Predict Models

The Pre-TACE-Predict model demonstrated a trend towards better performance when compared to the Post-TACE-Predict model, according to Harrell’s C index (0.649 versus (vs.) 0.634, respectively), Gönen & Heller’s K (0.629 vs. 0.625, respectively), Royston-Sauerbrei’s R^2^ (0.170 vs. 0.156, respectively), homogeneity (24.74 vs. 24.15%, respectively), discriminatory ability (22.83 vs. 21.34%, respectively), and Akaike information criterion (929.6383 vs. 930.2264, respectively) (Table 4).

The predictive performance of both the Pre-TACE-Predict and Post-TACE-Predict models was highest at the one-year time point (iAUC, 0.685 (95% CI 0.593–0.772); and iAUC, 0.659 (95% CI 0.580–0.742), respectively). However, there was no significant difference between the two models at any time point (one-year iAUC difference 0.026 (95% CI −0.071 to 0.109); two-year iAUC difference 0.032 (95% CI −0.045 to 0.100); and three-year iAUC difference 0.036 (95% CI −0.033 to 0.099)) (Table 5).

### 3.6. Comparison of the Predictive Performance among Models with and without Post-TACE Parameters

Among the models using only pre-treatment parameters, mHAP-II demonstrated the highest predictive performance for one-year iAUC (0.767 (95% CI 0.683–0.847)) compared to the Pre-TACE-Predict (0.685 (95% CI 0.593–0.772)) and HAP (0.718 (95% CI 0.627–0.809)) models. There was a significant difference between the performance of the Pre-TACE-Predict and mHAP-II models at the one-year time point (iAUC difference –0.082 (95% CI −0.170 to −0.003)). In addition, there was no significant difference between the Pre-TACE-Predict and HAP models at any time point (Table 6).

Among the models using the first TACE response, the SNACOR model demonstrated better predictive performance at the one-year iAUC (0.778 (95% CI 0.687–0.866)) compared to the Post-TACE-Predict model (0.659 (95% CI 0.580–0.742)). The SNACOR model was significantly more predictive at the one-year (iAUC difference, 0.119 (95% CI 0.008–0.223)) and three-year (iAUC difference, 0.084 (95% CI 0.001–0.161)) time points (Table 6).

## 4. Discussion

To the best of our knowledge, the present study was the first to externally validate the prognostic performance of the Pre-TACE-Predict and Post-TACE-Predict models compared with other TACE prognosis prediction models in an independent South Korean patient cohort with HCC. In the present study, the prognostic performances of the Pre-TACE-Predict and Post-TACE-prediction models were suboptimal, with one-year iAUCs of 0.685 and 0.659, respectively. When comparing model performances, Pre-TACE-Predict outperformed the Post-TACE-predict model in multiple analyses, including Harrell’s C index (0.6495 vs. 0.6314, respectively) and Gönen and Heller’s K (0.6273 vs. 0.6236, respectively). In addition, the simpler scoring models, such as the mHAP and SNACOR models, demonstrated comparable or even better performances, with a one-year iAUC of 0.767 and 0.778, respectively, when compared to the somewhat complex equations of the Pre-TACE-Predict and Post-TACE-Predict models. This is quite different from the findings of Han et al., in whose study cohort the Pre- and Post-TACE-Predict models performed significantly better than other conventional models.

Although the initial goal of our study was to validate the Pre- and Post-TACE-Predict models, the prediction performances of the models were not satisfactory. When compared to one another, the performance of the Pre-TACE-Predict model appeared to be greater than that of the Post-TACE-Predict model. This may be due to underlying differences in the model equations because the Pre-TACE-Predict model includes variables for etiology, which may have influenced the results in our HBV-dominant cohort. Additionally, both Pre- and Post-TACE-Predict models performed similarly or worse when compared to well-validated, simple prognosis prediction models. Although numerous variables are used in the pre- and post-TACE prediction models, it appears that the conventional tumor factors used in the simple scoring models are ultimately more effective in predicting survival. This finding is further supported by the fact that the performances of the Post-TACE-Predict and the SNACOR models were similar to those of models that did not incorporate the response after first TACE, indicating the significance of the conventional tumor factors used in the simple scoring models. Accordingly, it may be more clinically applicable for physicians to use simpler models, rather than the complex Pre- and Post-TACE-Predict models.

Our study had several strengths. First, the median OS of our cohort was 33.4 months, which was approximately equivalent to historical data from the international practice guidelines (i.e., approximately 40 months for patients with BCLC stage B) [21]. The survival timeline similarity with internationally accepted guidelines strengthens the generalizability of our results. In contrast, a cohort study by Han et al. established that the Pre-Predict and Post-TACE-Predict models had a median OS of 19.9 months. A significant difference in tumor characteristics between the two studies may explain the different survival rates. In fact, the median tumor size in our cohort was 2.9 cm, which was smaller than that in the cohort studied by Han et al., which ranged from 3.0 to 8.5 cm, depending on the subgroup. At our institute, intermediate stage or advanced HCC patients with vascular invasion, significant tumor burden of more than 10 cm, or infiltrative HCC were more likely to be treated with transarterial radioembolization [22] or liver-directed concurrent chemoradiotherapy [23]. Consequently, TACE has been frequently applied to patients with with nodular HCC presenting with relatively lower heavy tumor burden at our institute. Our therapeutic strategy may have affected the prognostic accuracy of the Pre- and Post-TACE-Predict models, which rely on variables such as vascular invasion and bilirubin, which are usually more strongly associated with patients with more advanced HCC.

Furthermore, our study highlights the need for updated guidelines to guide TACE treatment. The patients in our study, classified as BCLC stage B, were highly heterogeneous. Previous studies have proposed sub-classification of BCLC stage B HCC for detailed prognostication and corresponding treatment [24]. Bolondi et al. [25] proposed four sub-stages (B1–B4) with the addition of portal vein thrombosis and Child–Pugh score. Validation studies investigating the sub-classification reported statistically significant median survival differences between sub-stages, strengthening the need for updated guidelines and therapies for BCLC stage B. Although more evidence is required, patients determined as being early stage B should be down staged and recommended curative treatment if possible. Furthermore, patients determined to have high-risk BCLC stage B and poor responders to TACE should be switched to targeted systemic therapies [26,27]. Additionally, although patients in our cohort had a smaller tumor burden, only 2% were determined to be stage 1 in the Pre-TACE-Predict models, which could indicate that the Pre-TACE-model is not adequate for identifying low-risk HCC patients. Conversely, risk models, such as HAP and mHAP-II, exhibited a more even distribution between low- and high-risk patients in our study, which may support the potential benefit of these prognosis prediction models in determining treatment strategies for low-risk patients.

We also acknowledge several issues that remain unresolved. First, due to the retrospective nature of our study, the decision to perform TACE according to strict guidelines was not possible. In fact, in our study, patients who were diagnosed with BCLC stage C underwent TACE treatment. Thus, due to the tendency to use TACE outside of the BCLC stage B criteria, we believe that a large-scale validation and comparison study of the pre- and post-TACE prediction models with BCLC stage sub-analysis is necessary. Second, because all study participants were recruited from one institute, further studies are necessary to determine the applicability of the models to other institutes or countries. Third, the results could be strengthened by analysis of additional radiological or serological biomarkers that were not collected in our study. For example, additional analysis using the lectin Lens culinaris agglutinin binding glycoform of AFP (AFP-L3), derived only from cancer cells, which has been considered specific to HCC [28,29] or diffusion-weighted imaging, which is effective in tumor response assessment after TACE [30,31,32], may provide new insights into the outcomes and response to TACE. Finally, the overall sample size of our study was not large. Thus, potential statistical errors may have impacted the results. In addition, the number of patients in the Pre-TACE-Predict model group 1 was low. This may have been due to the high percentage of patients with underlying HBV infection as well as high initial AFP levels. As such, further large-scale studies are necessary to validate our findings.

## 5. Conclusions

There was no significant difference between the Pre- and Post-TACE prediction models in HCC patients. Additionally, simple scoring prognosis prediction models, such as mHAP-II and SNACOR, performed similarly to or better than the Pre- and Post-TACE-Predict models in our study. When considering the ease of application and better performance, models using a point system may be preferred over the Pre- and Post-TACE-Predict models in patients with HCC.

## Figures and Tables

**Table 1:** Calculation of HAP, mHAP-II, SNACOR scores and their risk stratification.

Models Based upon Only Pre-Treatment Factors			Models Based upon 1st TACE Response	
Variables	HAP score *	mHAP-II score *	Variables	SNACOR score *
Tumor size (>7 cm)	1	1	Tumor size (≥5 cm)	1
Tumor number (≥2)	N/A	1	Tumor number (≥4)	2
AFP (>400 ng/mL)	1	1	AFP (>400 ng/mL)	3
Total bilirubin (>0.9 mg/dL)	1	1	Child-Pugh class B	1
Serum albumin (<3.6 g/dL)	1	1	Objective response (SD or PD)	3

* HAP, mHAP-II, SNACOR scores are calculated as the sum of score allocated to each factor. Risk stratification by each model was as follows; A (0), B (1), C (2), and D (≥2) by HAP score, A (0), B (1), C (2), and D (≥2) by mHAP score, and low—(0~2), intermediate—(3~6) and high—(>7) risk by SNACOR score. TACE, transarterial chemoembolization; HAP, hepatoma arterial-embolization prognostic; mHAP-II, modified HAP-II; N/A, not applicable; AFP, alpha-fetoprotein; SNACOR, tumor Size and Number baseline Alpha-fetoprotein Child-Pugh and Objective radiological Response; SD, stable disease; PD, progressive disease.

**Table 2:** Baseline characteristics of the study population (*n* = 187).

Variables	Values
Demographic variables	
Age, years	59 (53–66)
Male gender	136 (72.7)
Etiology	
HBV/HCV/others	127 (67.9)/31 (16.6)/29 (15.5)
Liver cirrhosis Child-Pugh class	128 (66.8)
A/B	174 (93.0)/13 (7.0)
ALBI grade	
1/2/3	105 (56.1)/81 (43.3)/1 (0.5)
Tumor variables	
BCLC stage	
A/B/C	89 (47.6)/84 (44.9)/14 (7.5)
Tumor size, cm	2.9 (1.9–5.1)
Tumor number	2.0 (1.0–3.0)
Unifocal/Multifocal	93 (49.7)/94 (50.3)
Segmental PVT invasion	14 (7.5)
Laboratory variables	
Alpha-fetoprotein, ng/mL	136.1 (24.2–541.9)
Total bilirubin, mg/dL	0.7 (0.5–1.0)
Serum albumin, g/dL	4.0 (3.5–4.4)
Aspartate aminotransferase, IU/L	49 (35–76)
Prothrombin time, INR	1.05 (0.98–1.12)
Serum creatinine, mg/dL	0.9 (0.8–1.0)
Prognosis prediction models	
Pre-TACE model	
1/2/3/4	4 (2.1)/40 (21.4)/78 (41.7)/65 (34.8)
HAP score	
A/B/C/D	61 (32.6)/77 (41.2)/39 (20.9)/10 (5.3)
Modified HAP-II score	
A/B/C/D	34 (18.2)/64 (34.2)/60 (32.1)/29 (15.5)

Variables are expressed as median (interquartile range) or n (%); HBV, hepatitis B virus; HCV, hepatitis C virus; ALBI, albumin-bilirubin; BCLC, Barcelona clinic liver cancer; PVT, Portal vein thrombosis; INR, international normalized ratio; TACE, transarterial chemoembolization; HAP, hepatoma arterial-embolisation prognostic.

**Table 3:** Treatment response after the first TACE and risk assessment.

Variables	*n* (%)
Treatment response	
Complete response	28 (15.0)
Partial response	81 (43.3)
Stable disease	41 (21.9)
Progressive disease	37 (19.8)
Risk assessment	
Post-TACE score	
1/2/3/4	31 (16.6)/51 (27.3)/27 (14.4)/78 (41.7)
SNACOR score	
Low/Intermediate/High-risk	98 (52.4)/80 (42.8)/9 (4.8)

TACE, transarterial chemoembolization; SNACOR, tumor Size and Number baseline Alpha-fetoprotein Child-Pugh and Objective radiological Response.

**Table 4:** Performance of Pre- and Post-TACE score.

Statistical Parameters	Pre-TACE Score	Post-TACE Score	Difference in Values between Models
Harrell’s C index	0.6494 (0.5917, 0.6994)	0.6343 (0.5784, 0.6836)	0.0151 (−0.0451, 0.0712)
Gönen & Heller’s K	0.6289 (0.5791, 0.6753)	0.6250 (0.5785, 0.6685)	0.0039 (−0.0554, 0.0589)
Royston-Sauerbrei’s R2	0.1699 (0.0644, 0.2924)	0.1560 (0.0617, 0.2673)	0.0138 (−0.1318, 0.1471)
Homogeneity	24.74	24.15	N/A
Discriminatory ability	22.83	21.34	N/A
Akaike information criterion	929.6383	930.2264	N/A

TACE, transarterial chemoembolization; SE, standard Error; N/A, not applicable.

**Table 5:** Comparison between Pre- and Post-TACE-Predict models.

Time Point	Death/Number of Patients Analyzed	iAUC (95% CI)		Difference in iAUCs
		Pre-TACE-Predict	Post-TACE-Predict	
1 year	25/187	0.685 (0.593, 0.772)	0.659 (0.580, 0.742)	0.026 (−0.071, 0.109)
2 years	42/187	0.673 (0.599, 0.748)	0.641 (0.572, 0.709)	0.032 (−0.045, 0.100)
3 years	62/187	0.659 (0.597, 0.719)	0.624 (0.564, 0.688)	0.036 (−0.033, 0.099)

TACE, transarterial chemoembolization; iAUC, integrated areas under the receiver-operating curve; CI, confidence interval.

**Table 6:** Predictive performance of prognosis prediction models from the baseline and after the 1st TACE.

Time Point	Death/Total Patients	iAUC (95% CI)			Difference in iAUCs *	Difference in iAUCs **
		From the Baseline				
		Pre-TACE-Predict	HAP	mHAP-II		
1 year	25/187	0.685 (0.593, 0.772)	0.720 (0.627, 0.809)	0.767 (0.683, 0.847)	−0.035 (−0.146, 0.065)	−0.082 (−0.170, −0.002)
2 year	42/187	0.673 (0.599, 0.748)	0.652 (0.578, 0.724)	0.718 (0.652, 0.785)	0.026 (−0.069, 0.107)	−0.044 (−0.117, 0.031)
3 year	62/187	0.659 (0.597, 0.719)	0.634 (0.571, 0.695)	0.703 (0.647, 0.757)	0.025 (−0.045, 0.097)	−0.043 (−0.105, 0.016)
Time point	Death/total patients	After the 1st TACE			Difference in iAUCs	
		Post-TACE-Predict		SNACOR		
1 year	25/187	0.659 (0.580, 0.742)		0.778 (0.687, 0.866)	0.119 (0.008, 0.223)	
2 year	42/187	0.641 (0.572, 0.709)		0.698 (0.622, 0.773)	0.057 (−0.035, 0.152)	
3 year	62/187	0.624 (0.564, 0.688)		0.707 (0.646, 0.770)	0.084 (0.001, 0.161)	

* Difference for comparison between Pre-TACE-Predict and HAP score; ** Difference for comparison between Pre-TACE-Predict and mHAP-II score; HAP, hepatoma arterial-embolization prognostic; mHAP-II, modified HAP-II; TACE, transarterial chemoembolization; iAUC, integrated areas under the receiver-operating curve; CI, confidence interval.

## Data Availability

The data presented in this study are available on request from the corresponding author. The data are not publicly available due to privacy or ethical reason.

## References

[1] El-Serag H.B. (2012). Epidemiology of Viral Hepatitis and Hepatocellular Carcinoma. Gastroenterology.

[2] Lee E.W., Khan S. (2017). Recent advances in transarterial embolotherapies in the treatment of hepatocellular carcinoma. Clin. Mol. Hepatol..

[3] Dika I.E., Abou-Alfa G.K. (2017). Treatment options after sorafenib failure in patients with hepatocellular carcinoma. Clin. Mol. Hepatol..

[4] Kim J.H., Sinn D.H., Shin S.W., Cho S.K., Kang W., Gwak G.-Y., Paik Y.-H., Lee J.H., Koh K.C., Paik S.W. (2017). The role of scheduled second TACE in early-stage hepatocellular carcinoma with complete response to initial TACE. Clin. Mol. Hepatol..

[5] Llovet J.M., Real M.I., Montaña X., Planas R., Coll S., Aponte J., Ayuso C., Sala M., Muchart J., Solà R. (2002). Arterial embolisation or chemoembolisation versus symptomatic treatment in patients with unresectable hepatocellular carcinoma: A randomised controlled trial. Lancet.

[6] Lo C.-M., Ngan H., Tso W.-K., Liu C.-L., Lam C.-M., Poon R.T., Fan S.-T., Wong J. (2002). Randomized controlled trial of transarterial lipiodol chemoembolization for unresectable hepatocellular carcinoma. Hepatology.

[7] Bruix J., Sherman M. (2011). Management of hepatocellular carcinoma: An update. Hepatology.

[8] European Association For The Study Of The Liver (2012). Easl-eortc clinical practice guidelines: Management of hepatocellular carcinoma. J. Hepatol..

[9] Baek M.Y., Yoo J.-J., Jeong S.W., Jang J.Y., Kim Y.K., Jeong S.O., Lee S.H., Kim S.G., Cha S.-W., Kim Y.S. (2019). Clinical outcomes of patients with a single hepatocellular carcinoma less than 5 cm treated with transarterial chemoembolization. Korean J. Intern. Med..

[10] Lee D., Lee H.C., An J., Shim J.H., Kim K.M., Lim Y.-S., Chung Y.-H., Lee Y.S. (2018). Comparison of surgical resection versus transarterial chemoembolization with additional radiation therapy in patients with hepatocellular carcinoma with portal vein invasion. Clin. Mol. Hepatol..

[11] Lee J.S., Kim B.K., Kim S.U., Park J.Y., Ahn S.H., Seong J.S., Han K.H., Kim D.Y. (2020). A survey on transarterial che-moembolization refractoriness and a real-world treatment pattern for hepatocellular carcinoma in korea. Clin. Mol. Hepatol..

[12] Trevisani F., Golfieri R. (2016). Lipiodol transarterial chemoembolization for hepatocellular carcinoma: Where are we now?. Hepatology.

[13] Kim D.S., Lim T.S., Jeon M.Y., Kim B.K., Park J.Y., Kim D.Y., Ahn S.H., Han K.-H., Baatarkhuu O., Kim S.U. (2019). Transarterial Chemoembolization in Treatment-Naïve and Recurrent Hepatocellular Carcinoma: A Propensity-Matched Outcome Analysis. Dig. Dis. Sci..

[14] Kadalayil L., Benini R., Pallan L., O’Beirne J., Marelli L., Yu D., Hackshaw A., Fox R., Johnson P., Burroughs A.K. (2013). A simple prognostic scoring system for patients receiving transarterial embolisation for hepatocellular cancer. Ann. Oncol..

[15] Park Y., Kim S.U., Kim B.K., Park J.Y., Kim D.Y., Ahn S.H., Park Y.E., Park J.H., Lee Y.I., Yun H.-R. (2016). Addition of tumor multiplicity improves the prognostic performance of the hepatoma arterial-embolization prognostic score. Liver Int..

[16] Kim B.K., Shim J.H., Kim S.U., Park J.Y., Kim D.Y., Ahn S.H., Kim K.M., Lim Y.-S., Han K.-H., Lee H.C. (2016). Risk prediction for patients with hepatocellular carcinoma undergoing chemoembolization: Development of a prediction model. Liver Int..

[17] Han G., Berhane S., Toyoda H., Bettinger D., Elshaarawy O., Chan A.W.H., Kirstein M., Mosconi C., Hucke F., Palmer D. (2020). Prediction of survival among patients receiving transarterial chemoembolization for hepatocellular car-cinoma: A response-based approach. Hepatology.

[18] Lencioni R., Llovet J.M. (2010). Modified RECIST (mRECIST) Assessment for Hepatocellular Carcinoma. Semin. Liver Dis..

[19] Royston P., Altman D.G. (2013). External validation of a Cox prognostic model: Principles and methods. BMC Med. Res. Methodol..

[20] Steyerberg E.W., Vickers A.J., Cook N.R., Gerds T., Gonen M., Obuchowski N., Pencina M.J., Kattan M.W. (2010). Assessing the performance of prediction models: A framework for traditional and novel measures. Epidemiology.

[21] European Association For The Study Of The Liver (2018). Easl clinical practice guidelines: Management of hepatocellular carcinoma. J. Hepatol..

[22] Kim Y., Lee H.A., Lee J.S., Jeon M.Y., Kim B.K., Park J.Y., Kim D.Y., Ahn S.H., Um S.H., Seo Y.S. (2021). Association between curative treatment after transarterial radioembolization and better survival outcomes in patients with hepato-cellular carcinoma. Cancer Investig..

[23] Han S., Lee H.W., Park J.Y., Kim S.U., Kim D.Y., Ahn S.H., Han K.-H., Seong J., Won J.Y., Han D.H. (2020). Appraisal of Long-Term Outcomes of Liver-Directed Concurrent Chemoradiotherapy for Hepatocellular Carcinoma with Major Portal Vein Invasion. J. Hepatocell. Carcinoma.

[24] Golfieri R., Bargellini I., Spreafico C., Trevisani F. (2019). Patients with Barcelona Clinic Liver Cancer Stages B and C Hepatocellular Carcinoma: Time for a Subclassification. Liver Cancer.

[25] Bolondi L., Burroughs A., Dufour J.F., Galle P.R., Mazzaferro V., Piscaglia F., Raoul J.L., Sangro B. (2012). Heterogeneity of patients with intermediate (bclc b) hepatocellular carcinoma: Proposal for a subclassification to facilitate treatment deci-sions. Semin. Liver Dis..

[26] Ogasawara S., Ooka Y., Koroki K., Maruta S., Kanzaki H., Kanayama K., Kobayashi K., Kiyono S., Nakamura M., Kanogawa N. (2020). Switching to systemic therapy after locoregional treatment failure: Definition and best timing. Clin. Mol. Hepatol..

[27] Torimura T., Iwamoto H. (2021). Optimizing the management of intermediate-stage hepatocellular carcinoma: Current trends and prospects. Clin. Mol. Hepatol..

[28] Spangenberg H.C., Thimme R., Blum H.E. (2006). Serum Markers of Hepatocellular Carcinoma. Semin. Liver Dis..

[29] Tsuchiya N., Sawada Y., Endo I., Saito K., Uemura Y., Nakatsura T. (2015). Biomarkers for the early diagnosis of hepatocellular carcinoma. World J. Gastroenterol..

[30] Lee J.M., Yoon J.-H., Kim K.W. (2012). Diagnosis of Hepatocellular Carcinoma: Newer Radiological Tools. Semin. Oncol..

[31] Bruegel M., Holzapfel K., Gaa J., Woertler K., Waldt S., Kiefer B., Stemmer A., Ganter C., Rummeny E.J. (2008). Character-ization of focal liver lesions by adc measurements using a respiratory triggered diffusion-weighted single-shot echo-planar mr imaging technique. Eur. Radiol..

[32] Parikh T., Drew S.J., Lee V.S., Wong S., Hecht E.M., Babb J.S., Taouli B. (2008). Focal Liver Lesion Detection and Characterization with Diffusion-weighted MR Imaging: Comparison with Standard Breath-hold T2-weighted Imaging. Radiology.

